# The effect of extrinsic mortality on genome size evolution in prokaryotes

**DOI:** 10.1038/ismej.2016.165

**Published:** 2016-12-06

**Authors:** Piotr Bentkowski, Cock van Oosterhout, Ben Ashby, Thomas Mock

**Affiliations:** 1 School of Environmental Sciences, University of East Anglia, Norwich Research Park, Norwich, UK; 2 Institute of Environmental Biology, Faculty of Biology, Adam Mickiewicz University, Poznań, Poland; 3 Department of Mathematical Sciences, University of Bath, Bath, UK; 4 Department of Integrative Biology, University of California, Berkeley, CA, USA

## Abstract

Mortality has a significant role in prokaryotic ecology and evolution, yet the impact of variations in extrinsic mortality on prokaryotic genome evolution has received little attention. We used both mathematical and agent-based models to reveal how variations in extrinsic mortality affect prokaryotic genome evolution. Our results suggest that the genome size of bacteria increases with increased mortality. A high extrinsic mortality increases the pool of free resources and shortens life expectancy, which selects for faster reproduction, a phenotype we called ‘scramblers'. This phenotype is realised by the expansion of gene families involved in nutrient acquisition and metabolism. In contrast, a low mortality rate increases an individual's life expectancy, which results in natural selection favouring tolerance to starvation when conditions are unfavourable. This leads to the evolution of small, streamlined genomes (‘stayers'). Our models predict that large genomes, gene family expansion and horizontal gene transfer should be observed in prokaryotes occupying ecosystems exposed to high abiotic stress, as well as those under strong predator- and/or pathogen-mediated selection. A comparison of genome size of cyanobacteria in relatively stable marine versus more turbulent freshwater environments corroborates our predictions, although other factors between these environments could also be responsible.

## Introduction

The genome size varies widely across species (Koonin and Wolf, 2008; Elliott and Gregory, 2015) and does not strongly correlate with the complexity of the organisms, that is, the ‘C-value paradox' (Gregory, 2001; Elliott and Gregory, 2015). Both selective and neutral processes are thought to govern this variation, and a considerable proportion of it is explained by differences in the number of repetitive elements, such as transposons (Lynch, 2007). There is also variation in the size of multigene families, and closely related species occupying similar environments can vary significantly in their copy number variation (CNV), which suggests that contrasting selective processes may be operating driving the contraction or expansion of gene families (Greenblum *et al.*, 2015). Previously, we have shown that a greater variability of one environmental selection pressure results in genomes with a larger number of genes, and those environmental perturbations are more effectively buffered by populations with relatively large genomes (Bentkowski *et al.*, 2015).

However, in the process of biological evolution, organisms are forced to compromise between challenges posed by several different selection pressures. Virus resistance in bacteria is a well-described trade-off involving the assessment of fitness costs. For instance, phage-resistant clones of the soil bacterium *Pseudomonas fluorescens* had ~36% lower relative fitness versus sensitive clones when the phage was absent (Gómez and Buckling, 2011). In the marine cyanobacterium *Synechococcus*, the cost of resistance, if measurable, resulted in approximately 20% reduction of fitness (Lennon *et al.*, 2007). Cost of resistance helps to shape microbial communities especially in aquatic environments (Suttle, 2007; Parsons *et al.*, 2012; Sime-Ngando, 2014) by selectively increasing mortality of bacteria that are more susceptible (or less tolerant) to viruses but only when pathogens are present. Without pathogens, cost of resistance is a burden and selection would favour individuals with less resistance but with more resources for coping with other environmental challenges. A similar conflict in selection pressures has been proposed to explain the CNV in vertebrate immune genes and is known as the accordion model of multigene evolution (Klein *et al.*, 1993). The accordion model poses that immune gene families expand in parasite-rich environments and, conversely, that they contract when parasite selection pressures are modest. Other important factors imposing different levels of stress and mortality on bacterial communities include negative allelopathy (that is, detrimental interspecific biochemical interactions), predation, harmful radiation and toxins (Omelchenko *et al.*, 2005; Weinbauer *et al.*, 2007; Hibbing *et al.*, 2010).

Bacteria have limited amounts of nutrients and energy to survive these challenges and to reproduce. The strength of the various selection pressures on nutrient acquisition and allocation fluctuate depending on biotic and abiotic environmental conditions. Here we apply two theoretical frameworks: a mathematical model and an agent-based model (ABM; Bentkowski *et al.*, 2015) to untangle the effect of extrinsic mortality caused by various stresses on the evolution of genome size in prokaryotic microbial populations. We compare our theoretical predictions on genome size evolution with differences in genome sizes of 63 species of cyanobacteria: 30 marine and 33 freshwater species.

## Materials and methods

The effect of extrinsic mortality on genome size evolution was estimated through a combination of mathematical modelling and agent-based simulations. Initially, we used an algebraic model to generate a qualitative prediction for the impact of the mortality rate on genome size evolution. Results from this model were verified and extended by agent-based simulations, enabling us to explore genome size evolution under very general conditions as well as more realistic ecological scenarios. The algebraic model is based on the following set of ordinary differential Equations:


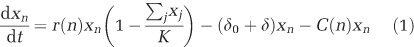


where *x*_*n*_ is the density of cells with genome size *n*, *r*(*n*) is the rate at which nutrients are absorbed—cells with larger genomes are able to uptake nutrients at a higher rate than cells with smaller genomes (that is, *r*(*n*) is an increasing function of genome size, *n*)—leading to asexual reproduction, *K* is the maximum density of the population, *δ*_0_ is the base mortality rate (for example, owing to ageing), *δ* is an extrinsic mortality rate (for example, owing to environmental factors) and *C*(*n*) is a cost associated with genome size. Protein synthesis is considered to require most of the energy and nutrients in cells (Harold, 1986) and therefore has been considered a significant fitness cost for increasing the number of genes in genomes. It also has been shown that, regardless of their function, large and complex proteins have a higher fitness cost as they require more energy and nutrients compared with their smaller, less complex counterparts (Tomala and Korona, 2013). Thus we assume there is a metabolic cost associated with genome size proportional to *n*+*n*^2^, as it has been demonstrated that an increase in genome size is directly proportional to the number of metabolic genes but grows quadratically with the number of regulatory genes (genome scaling law; van Nimwegen, 2003; Molina and van Nimwegen, 2008; Koonin 2011). We therefore set *C*(*n*)=*cn* (*n*+1), where *c* scales with the strength of the fitness cost. We investigate the evolutionary dynamics of the algebraic model using evolutionary invasion analysis, whereby mutations are rare and have small effects (weak selection; Geritz *et al.*, 1998). Hence, new mutants are phenotypically similar to the resident population (the trait under selection is continuous) and there is a separation of ecological and evolutionary timescales. These assumptions are relaxed in population-level simulations of the algebraic model, whereby mutants are introduced before the system reaches equilibrium and genome size is limited to integer values. These simulations are used to expand on the mathematical analysis.

The purpose of the algebraic model is to generate a simple qualitative prediction for the impact of the mortality rate on the evolution of genome size. However, this model cannot fully capture the complexity of real ecological systems, such as environmental variation, fluctuating resources, cell life cycles and specialisation on different resources. We therefore test the generality of the predictions derived from the algebraic model in a more realistic agent-based model (Bentkowski *et al.*, 2015). The agent-based simulations represent a population that is composed of approximately 3200 individual prokaryotic cells, each containing initially between 40 and 60 genes which values were set randomly. The number and values of genes (maximal uptake efficiency and position of the maximum on environmental axis) is subject to evolution. The size of the population was limited by the total amount of resource available in the environment *R*_env_ (equivalent of the environment's carrying capacity) and the size can change as the population evolves. Environmental conditions are given in the ABM as one trait changing in a random bounded walk within the range *x* in [−1, +1]. By regulating the maximal permitted value of environmental change (called the turbulence level *T* in this study) from one model iteration to the next one, we can regulate environmental variability and hence the span of environmental conditions affecting the individuals simulating various abiotic stress levels. The impact of variation in turbulence on genome size evolution was studied in a previous paper (Bentkowski *et al.*, 2015), and here we use a set level of turbulence (that is, high turbulence, *T*=0.25). High turbulence eliminates genome size fluctuations observed at low levels of turbulence (see Bentkowski *et al.*, 2015) and results in large, stable genomes. Hence, this enables us to investigate the effects of *δ*, the probability of random death, without the confounding effects caused by low turbulence. We thus simulate sources of mortality other than starvation resulting from mismatch between genotype and current environmental condition influencing nutrient uptake (for example, mortality owing to infections, predation, harmful radiation, chemical damage).

The effect of a single gene is represented as a Gaussian function over the environmental condition *x*, which dictates its uptake efficiency (a fraction of the maximal permitted value *τ* a cell can take from the environment in single iteration). Both the position of the maximum of the function along the *x* axis as well as the height of the function (limited to unity) are subject to evolve. However, the surface underneath the Gaussian function is fixed to prevent the evolution of ‘supergenes' (that is, single genes that maximise fitness across a wide environmental range). A genome is a collection of *n* metabolic genes (*n* is free to evolve) that bring a metabolic cost proportional to *n*+*n*^*2*^ as in the mathematical model above. At each time step, the change of internal resources depends on: (1) individual cell's genome shape (that is, the overall distribution of Gaussian functions of a cell's genes that describe its uptake efficiency, cf. Bentkowski *et al.*, 2015), (2) availability of resource, (3) cost proportional to the size of the cell's genome (*~n*+*n*^*2*^). If a cell's internal resource *R*_i_ will drop below survivor threshold *r*_min_, then the cell will die and its remaining internal resource pool *R*_i_ will be returned to the free environmental resource pool. In other words, these simulations model a closed system. In the ABM, nutrient uptake by cells does not depend directly on the number of genes as in the mathematical model (see *r*(*n*) in Equation 1), but it depends on possessing genes that maximise uptake under given environmental conditions.

There are three kinds of mutations affecting genome evolution in our artificial prokaryotic populations: deletions, duplications, and modifications of genes. Each type of mutation is occurring at the same probability. A deletion results in removal of a gene from a genome, a duplication results in a genome having two identical copies of a gene (for example, a cell will not benefit from this by doubling its intake in gene's optimum) and a modification is a change in height and position of the Gaussian-shaped uptake efficiency distribution.

The ABM is capable of: (a) reproducing genome streamlining under pressure of inter-cell competition in terms of resource uptake efficiency, and (b) rearrangement of the genome depending on amount of variability of environmental conditions (Bentkowski *et al.*, 2015). To estimate population-wide effects, we calculated the mean number of genes in a population averaged over all time steps at equilibrium. More details of the ABM can be found in Bentkowski *et al.* (2015) and documentation accompanying the source code (www.bitbucket.org/pbentkowski/genomesizeevolution).

To compare the output of our model with empirical data, we analysed the number of genes from 63 species of aquatic cyanobacteria with well-annotated genomes (Prabha *et al.*, 2016) occupying diverse environments. We compared genome sizes of freshwater and marine species. The majority of marine species with small genomes (*Prochlorococcus*) come from open ocean waters considered stable, low-nutrient environments (Christaki *et al.*, 1999; Sun and Blanchard, 2014) with lower concentration of viruses per millilitre in comparison to freshwater and costal habitats (Wommack and Colwell, 2000).

## Results

### Qualitative prediction

We begin by exploring the evolutionary dynamics of a very simple algebraic model (Equation 1). Assuming the population is initially monomorphic, the fitness of a rare mutant, *x*′, with genome size *n*′ is


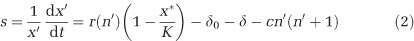


where x* is the non-trivial ecological equilibrium for the resident population with genome size *n*:


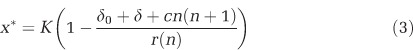


The population evolves in the direction of the selection gradient


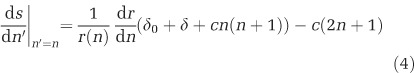


until a singular strategy, n*, is reached at 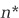. The singular strategy occurs when


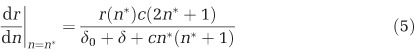


and is evolutionarily stable 
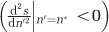
 provided we make the reasonable assumption that resource uptake and hence reproduction rate is a decelerating function of genome size 
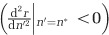
. For example, if we set *r*(*n*)=*bn*^1/2^, then the population evolves towards the evolutionarily stable strategy


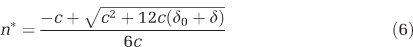


**Figure 1:**
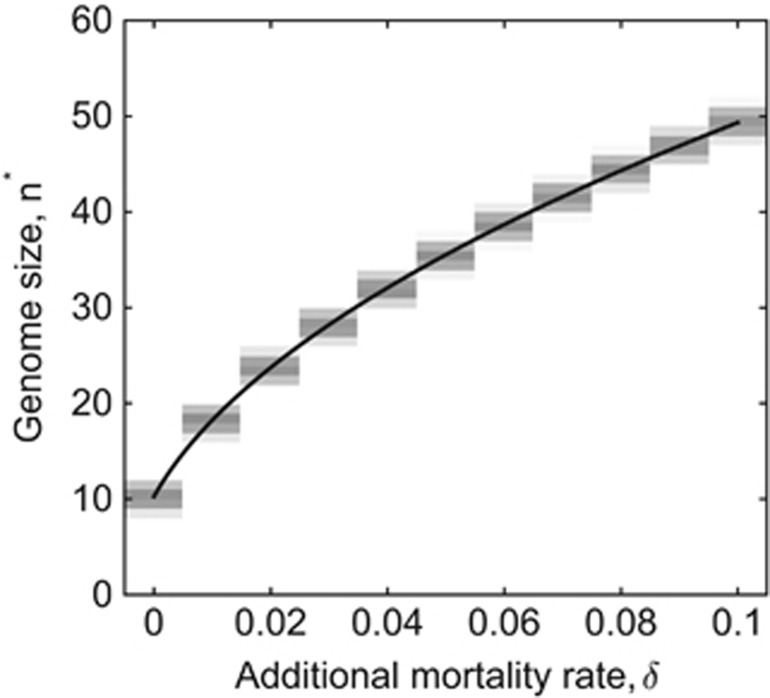
The evolution of genome size as a function of the additional mortality rate, as predicted by the algebraic model (black curve, Equation 6) and tested through population-level simulations (grey bars). The population-level simulations relax key assumptions of the mathematical analysis, namely, limiting genome size to integer values and removing the separation of ecological and evolutionary timescales leading to a mutation-selection balance. The shading of the grey bars corresponds to the average frequency of each genome size over the final 1000 iterations of each simulation, with *δ*=0, 0.01, …, 0.1. Other parameters: *b*=1, *c*=0.000014, *K*=10^6^ and *δ*_0_=0.005.

Thus our simple algebraic model predicts that optimal genome size increases with the mortality rate. Population-level simulations of the model match the analytic prediction (Figure 1). We test this general prediction in a more realistic ecological setting through agent-based simulations which relax many of the simplifying assumptions used in the above analysis.

### Agent-based simulations

**Figure 2:**
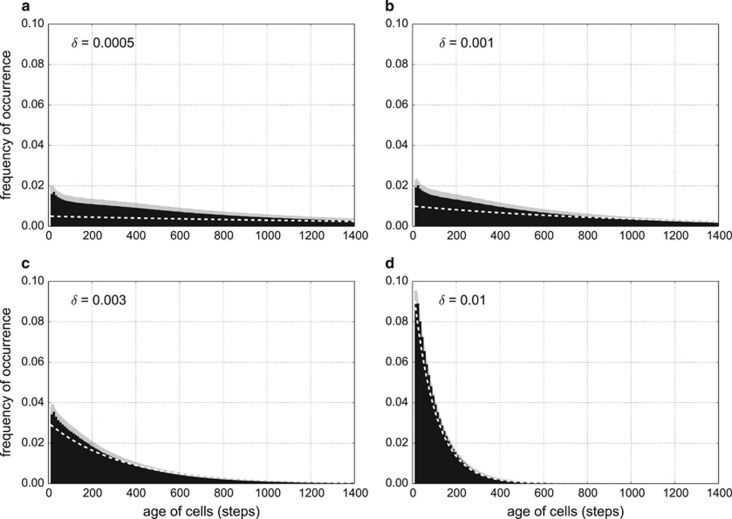
Age structure of the population in various random death regimes. Numbers on the left of the panels show the value of the random death factor *δ* ((**a**)—0.0005, (**b**)—0.001, (**c**)—0.003, (**d**)—0.01). Black bars indicate fraction of the population of a particular age, shaded area indicates the s.d. White dashed line is given by the equation of the exponential distribution: 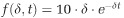 (Equation 7), where *t* is the time and 10 is a normalising term being the width of the histogram bins. All four runs have turbulence level *T*=0.25.

The extinction rate increases with increasing value of *δ*, the probability of random death (Supplementary Figure S1) and *δ* impacts the genome size independently of the turbulence level *T* (Supplementary Figure S2). The age structure of the population is given by the exponential distribution:


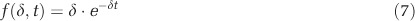


where *t* is the time and *δ* is the random death factor (extrinsic mortality). The expected lifespan of an individual is equal to 1/*δ*. Figure 2 shows that the age structure fits the exponential model very well when *δ* is fairly high (*δ*=0.01, Figure 2c). However, for *δ*⩽0.001, the exponential distribution no longer fits the observed age distribution, and there appears to be a significant excess of young individuals in the system (*δ*=0.0005 and 0.001, Figures 2a and b). This suggests that, besides random death, there must be another important source of mortality in these populations.

### Starvation resilience, competitive advantage and genome size

**Figure 3:**
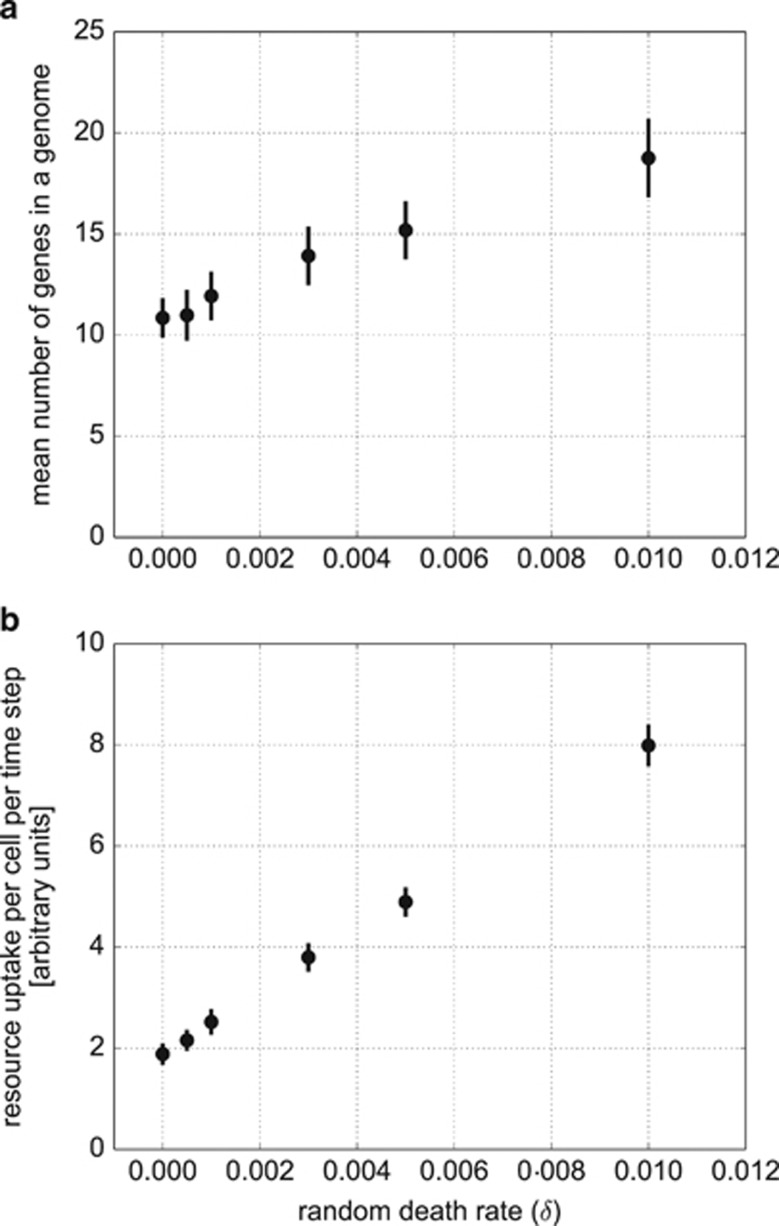
Model's sensitivity to changes in the values of the random death factor *δ*. (**a**) The mean number of genes at equilibrium as a function of the random death factor *δ* with s.d. (bars), (**b**) mean resource uptake per one cell in one time step with s.d. (includes only the cells that gained access to feeding queue: see Methods section). All runs had turbulence level *T*=0.25.

As the value of *δ* decreases, the grand mean number of genes also decreases. For example, without random death (*δ*=0) and high turbulence (*T*=0.25), the grand mean number of genes equals 11.4±0.9 (mean±s.d.). For moderate random death (*δ*=0.003), the grand mean number of genes is 13.9±1.5 (mean±s.d.), for high value of *δ*=0.010 it is 18.8±1.9 (Figure 3a).

**Figure 4:**
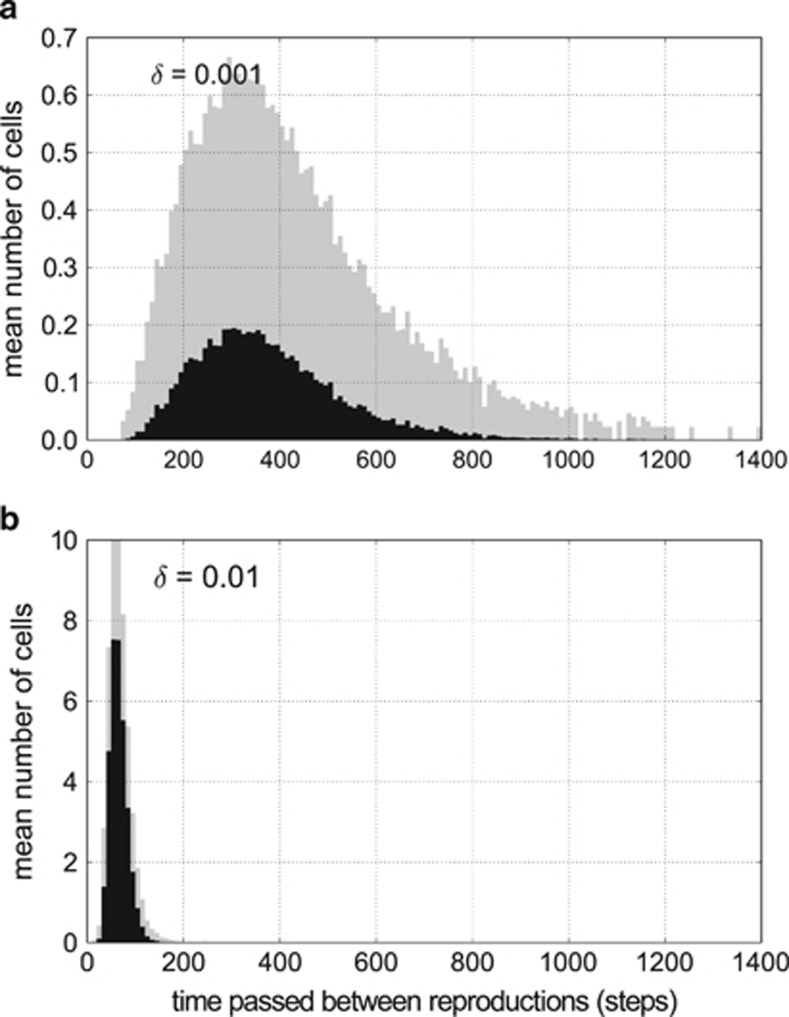
Histograms of the number of cells that reproduced after a given time since their last reproduction with extreme values of the random death factor: (**a**) low random death rate *δ*=0.001, (**b**) high death rate *δ*=0.01; black bars are the mean values per time step, shaded bars are the s.d. All values are averaged per one time step. Turbulence level was set to *T*=0.25.

**Figure 5:**
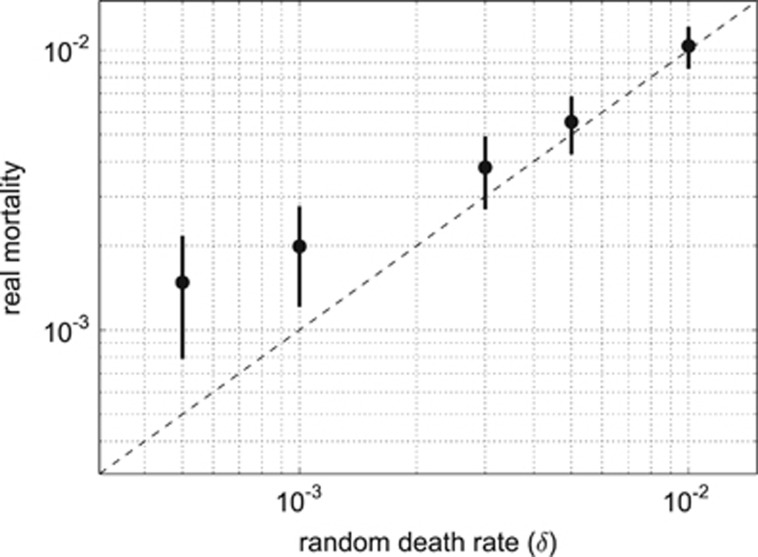
Comparison of the real mortality rates between simulations with different random death probabilities (turbulence level was *T*=0.25 for all runs). Dots represent a mean value for each model run calculated after the system has stabilized, the bars show the s.d. Dashed line is the equality line where all mortality is explained by the random death factor *δ*. Both axes are in the logarithmic scale. As the probability of random death increases, it becomes the dominant source of mortality in populations.

Interestingly, with an increased rate of random death, cells gain more resources per unit time; individuals that evolved in a low random death environment (*δ*=0.001) gained on average 2.85 resource units per time step, whereas in high death rate (*δ*=0.010) the gain equalled 7.98 resource units per time step (Figure 3b). This difference in resource uptake efficiency is explained by differences in genome size and metabolic rate in the two environments. Large genomes with higher metabolic rates evolved in high random death environments (Figure 3). Given that individuals with larger genomes require less time to accumulate sufficient resources to reproduce, the rate of reproduction is expedited, which offers an important selective advantage, particularly for individuals in a high random death environment. Indeed, it takes on average only 70.6 time steps for an average individual to reproduce in the high random death environment (*δ*=0.01), compared with 290.0 time steps in low random death conditions (*δ*=0.001) (Figure 4). By increasing genome size, individuals in the high random death environment not only reduce the risk of dying before reproduction but they also increase their competitiveness in resource-uptake efficiency. The relatively increased CNV enables these organisms (‘scramblers') to fully utilise the bounty of free resources available in the environment. In contrast, in the low random death environment, selection favours individuals that are tolerant to starvation, which is responsible for half of their mortality (Figure 5). These are the so-called ‘stayers', which are characterised by relatively smaller genome sizes that reduces metabolic maintenance costs associated with protein synthesis and translation.

### Model predictions versus empirical data

**Figure 6:**
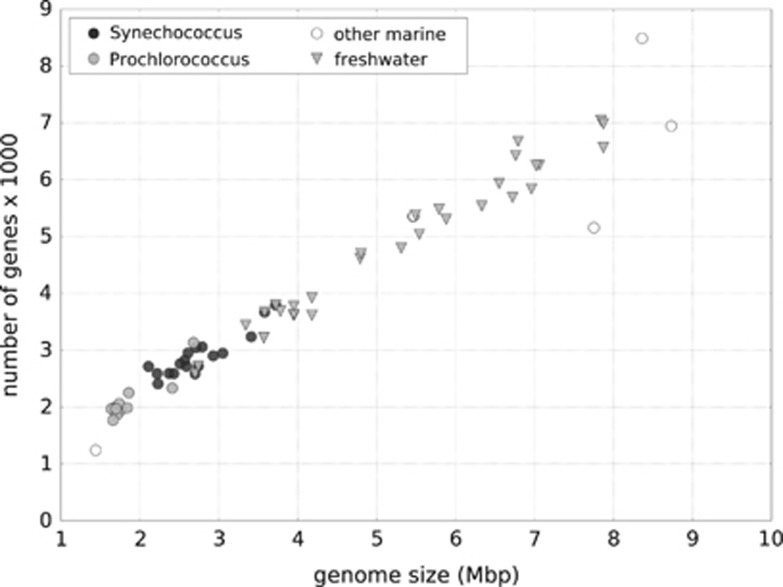
Comparison of genome sizes of aquatic cyanobacteria occupying two different habitats: marine (circles, 30 genomes), including: *Synechococcus* (• black circles), *Prochlorococcus* (• grey circles), other marine (○ empty circles); freshwater (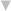 triangles, 33 genomes). The number of predicted protein-coding genes is significantly larger in the (turbulent) freshwater habitat than in the (more stable) marine habitat (Mann–Whitney test: *U*=354.0; *P*=0.027). Data are adapted from Prabha *et al.* (2016).

We compared our model predictions with gene numbers from 63 species of aquatic cyanobacteria that differ in their environmental preferences (Prabha *et al.*, 2016). Marine species, many of which occupy low-nutrient zones of the open ocean, tend to have relatively small genomes in comparison to freshwater species (Mann–Whitney test; *U*=354.0; *P=*0.027; Figure 6). It was shown that marine species have evolved less metabolic genes than cyanobacteria from more complex habitats (for example, freshwater and soil; Prabha *et al.,* 2016).

## Discussion

Our simple mathematical model showed that, under the assumption that cells with larger genomes are able to uptake nutrients at a higher rate than cells with smaller genomes (function *r*(*n*) in Equation 1), the genome size will grow in response to increased extrinsic mortality. The ABM simulations confirmed these results even when the dependency of nutrient uptake on genome size is replaced with a more realistic requirement such as cells with maximum nutrient uptake under a given environmental condition. In the mathematical model, higher mortality is compensated for by evolution of larger genomes with higher nutrient uptake leading to more cell divisions per unit time. The positive relationship between genome size and mortality will collapse by either having nutrient acquisition not in balance with genome size expansion or when the cost of genome maintenance becomes too large for the uptake mechanism to sustain the supply of nutrients. Under these unbalanced conditions, the genome size will reach the ‘van Nimwegen Limit' (Koonin and Wolf, 2008). However, ABM reveals a time constraint on the life expectancy as a second factor promoting increased nutrient uptake and faster reproduction (compare Figures 3b and 4). The simple mathematical model allows analytic tractability showing that (under assumptions discussed above) the solution we found is an evolutionary stable state (Maynard Smith, 1982). The more complex ABM allows for more realistic assumptions and the attribution of selection factors to individuals, which simulates evolutionary processes more realistically (Grimm, 1999).

A minimum set of metabolic genes is required for an individual to acquire sufficient resources to reproduce within its lifespan (Martínez-Cano *et al.*, 2015), and this sets the lower limit on genome streamlining. Although potentially immortal, the window of opportunity to reproduce is time limited in most prokaryotes because of a large number of environmental constraints and stress factors causing mortality. This time constraint imposes a trade-off on resource acquisition. Here we show that, in an environment with high random death rate (that emulates other sources of mortality than resource limitation only) and steady supply of nutrients, natural selection favours prokaryotes with large genome size and high CNV, which increases metabolic activity and accelerates the potential rate of reproduction. Importantly, this enables prokaryotes to rapidly utilise free resources when available and reduces the risk of dying without producing offspring. Furthermore, by increasing genome size and CNV, these prokaryotes (‘scramblers') are better able to utilise the bounty free resources available in the environment left by deceased conspecifics. On the other hand, in environments with low random death, selection favours ‘stayers', that is, individuals that are tolerant to starvation. These individuals have relatively small genome sizes, which reduce metabolic maintenance costs associated with protein synthesis and translation.

The efficiency and costs of metabolism underpinning reproduction are under strong selection pressure, especially in prokaryotes with large effective population sizes, which means that their evolution is less affected by genetic drift. Under these circumstances, natural selection tends to trim the size of genomes (Lynch, 2006; Giovannoni *et al.*, 2014). Additional costs are imposed by investing in defence mechanisms in hostile environments to enable reproduction before the end of the maximum expected lifespan. If the environment is lacking significant stress factors, inter-cell competition for resources becomes the main selection pressure, making defensive mechanisms and complex regulatory mechanisms costly burdens that get discarded in the course of evolution. This in turn results in genome streamlining and efficient, low-cost genomes.

Direct validation of our models with empirical data is difficult. Although there are some data on mortality of microbes, many studies focus on sources of mortality in isolation such as either predation (Christaki *et al.*, 1999; Liu *et al.*, 1995) or viral infections (Sime-Ngando, 2014; Tschitschko *et al.*, 2015), hence integrative approaches addressing the interactions between different sources of mortality are under-represented. Furthermore, growth experiments with microbes usually focus on comparing physiological abilities of different strains in the context of nutrient supply and interspecific competition (Lürling *et al.*, 2013; Bullerjahn and Post, 2015), but only a few studies include the analysis of reproductive strategies and mortality. Thus, to properly validate our models with empirical data, integrative experiments would need to be conducted, which include the assessment of reproductive strategies, mortality and the impact of environmental conditions on the evolution of microbes. However, an indirect validation of our models is possible when considering the relationship between genome sizes and environmental conditions for prokaryotic populations. For example, marine cyanobacteria tend to have smaller genome sizes in comparison with those residing in more complex habitats (for example, freshwater and soil; Prabha *et al.*, 2016). Our comparison of 30 marine and 33 freshwater species showed significant genome size differences between marine (mainly *Synechococcus* and *Prochlorococcus*) and various freshwater cyanobacteria (Figure 6). The genus *Prochlorococcus*, characterised by relatively small genomes, is mostly occurring in low nutrient and stable open-ocean waters (Christaki *et al.*, 1999; Sun and Blanchard, 2014). Furthermore, open-ocean habitats have much less virus-like particles per millilitre than freshwater habitats (Wommack and Colwell, 2000), hence contributing to lower pathogen pressure. Although marine species with very large genomes do exist, among them are species with unusual properties such as *Acaryochloris marina* MBIC11017, which contains chlorophyll *d* and is characterised by special adaptations (Swingley *et al.*, 2008). *Rivularia* sp. PCC 7116 is another example of a marine cyanobacterium with a relatively large genome but it is closely related to freshwater species (Marco *et al.*, 2012).

In summary, how natural selection will shape the genome size of prokaryotes partly depends on whether reproduction and survival are constrained by time (that is, in an environment with high random mortality) or whether there are constraints in the amount of available nutrients. Our simulations showed, when time constrained, natural selection favours prokaryotes that are ‘scramblers', that is, individuals with large genome size and high CNV, which increases metabolic activity and accelerates the rate of reproduction under conditions of nutrient repletion. Such species may evolve different solutions to exploit the abundant resources, resulting in significant genome diversity. With low random death, the amount of available nutrients becomes limiting and intraspecific competition becomes stronger. Thus selection favours the evolution of ‘stayers', that is, individuals that are tolerant to starvation with relatively small genomes and low metabolic maintenance costs. This simple model can explain some of the variation in genome size observed in cyanobacteria that occur in different environments.
