# Supplementary material for: The effect of extrinsic mortality on genome size evolution in prokaryotes
Source: ISME J. 2016 Dec 6;11(4):1011–8. doi: 10.1038/ismej.2016.165 (PMC5364348; doi:10.1038/ismej.2016.165)
Supplement: Supplementary Material [file ismej2016165x1.pdf]

Supplementary Material

For values of the random death rate $\delta \geq 0.03$ simulated populations never survive to the end of the model runs. The higher the value of δ, the faster the extinction occurs (Figure S1).

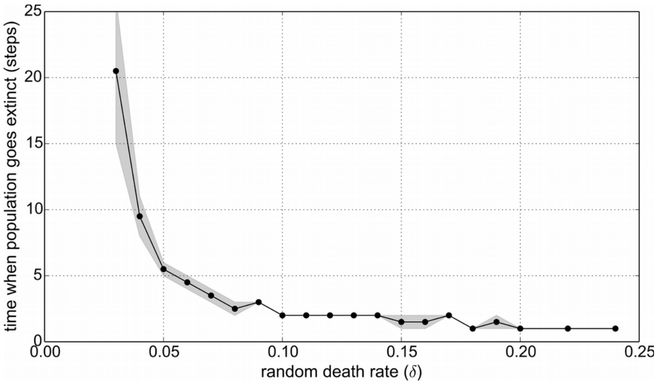

Fig. S1. High random death rate factor triggers extinction events and the higher is its value, the earlier extinction happens. Circle (•) represent the mean value of 4 runs, the shaded area is the SD. The death rate (δ) varied between [0, 0.24]; turbulence level was set to T = 0.01.

Higher random death rate (δ) increases the number of metabolic genes independently of the turbulence level (T) parameter (Figure S2). For low T, simulations have similar number of genes regardless of δ, but for T of moderate and high value δ has additional impact on gene number. Similar genome sizes for low T regardless of δ values are observed because there is no significant variability of the environment during expected life span of cells thus there is no selection for genomes able to acquire resources in a wide spectrum of environmental conditions. Every successful cell has to have a small number of the right kind of genes. For more discussion on how the level of turbulence T impacts gene number see: Bentkowski P, Oosterhout C van, Mock T. (2015). A model of genome size evolution for prokaryotes in stable and fluctuating environments. *Genome Biol Evol* **7**: 2344–2351.

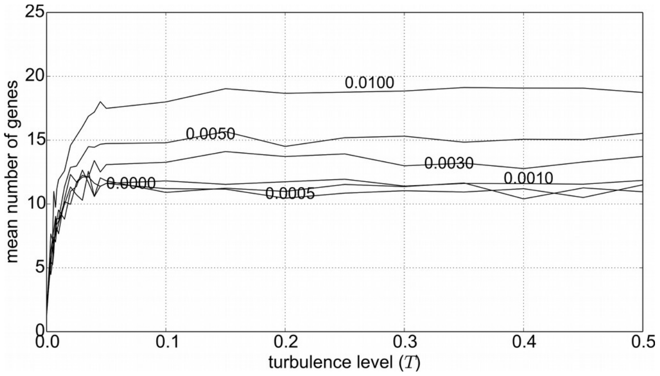

Fig. S2. Mean number of genes as function of turbulence level T shows model's sensitivity to changes in the values of the random death factor δ. Numbers above curves are the respective values of the random death factor δ. Uncertainty has been omitted for the sake of simplicity of the graph.

Organism Name	Abbreviation	Habitat	Genome Size (Mb)	No. of Plasmids	No. of Genes	% of COGs
Anabaena cylindrica PCC 7122	Ac_PCC_7122	Aquatic (Freshwater)	7.06	6	6258	49.65
Arthrospira platensis NIES-39	Ap_NIES_39	Aquatic (Freshwater)	6.79	0	6676	36.38
Anabaena sp. 90	As_90	Aquatic (Freshwater)	5.31	3	4797	49.78
Cyanobacterium aponinum PCC 10605	Ca_PCC_10605	Aquatic (Freshwater)	4.18	1	3614	57.39
Cyanobium gracile PCC 6307	Cg_PCC_6307	Aquatic (Freshwater)	3.34	0	3439	55.25
Chamaesiphon minutus PCC 6605	Cm_PCC_6605	Aquatic (Freshwater)	6.76	2	6427	46.65
Calothrix sp. PCC 6303	Cs_PCC_6303	Aquatic (Freshwater)	6.96	3	5840	48.89
Cyanothece sp. PCC 7424	Cs_PCC_7424	Aquatic (Freshwater)	6.55	6	5933	48.26
Cyanothece sp. PCC 7425	Cs_PCC_7425	Aquatic (Freshwater)	5.79	3	5481	51.51
Calothrix sp. PCC 7507	Cs_PCC_7507	Aquatic (Freshwater)	7.02	0	6250	51.6
Cyanothece sp. PCC 7822	Cs_PCC_7822	Aquatic (Freshwater)	7.84	6	7041	44.44
Cyanothece sp. PCC 8801	Cs_PCC_8801	Aquatic (Freshwater)	4.79	3	4615	53.52
Cyanothece sp. PCC 8802	Cs_PCC_8802	Aquatic (Freshwater)	4.8	4	4697	52.74
Dactylococcopsis salina PCC 8305	Ds_PCC_8305	Aquatic (Freshwater)	3.78	0	3685	53.32
Gloeocapsa sp. PCC 7428	Gs_PCC_7428	Aquatic (Freshwater)	5.88	4	5304	59.05
Halothece sp. PCC 7418	Hs_PCC_7418	Aquatic (Freshwater)	4.18	0	3920	58.14
Leptolyngbya sp. JSC-1	Ls_JSC_1	Aquatic (Freshwater)	7.87	1	6566	50.05
Leptolyngbya sp. JSC-1	Ls_JSc_1	Aquatic (Freshwater)	7.87	1	6986	46.71
Nostoc azollae 0708	Na_0708	Aquatic (Freshwater)	5.49	2	5379	37.91
Nostoc sp. PCC 7107	Ns_PCC_7107	Aquatic (Freshwater)	6.33	0	5538	53.81
Nostoc sp. PCC 7524	Ns_PCC_7524	Aquatic (Freshwater)	6.72	2	5688	54.36
Stanieria cyanosphaera PCC 7437	Sc_PCC_7437	Aquatic (Freshwater)	5.54	5	5041	53.78
Synechococcus elongatus PCC 6301	Se_PCC_6301	Aquatic (Freshwater)	2.7	0	2585	62.17
Synechococcus elongatus PCC 7942	Se_PCC_7942	Aquatic (Freshwater)	2.74	1	2719	60.35
Synechocystis sp. GT-S, PCC 6803	Ss_GT_S_PCC_6803	Aquatic (Freshwater)	3.57	0	3220	65.12
Synechococcus sp. PCC 6312	Ss_PCC_6312	Aquatic (Freshwater)	3.72	1	3794	52.4
Synechocystis sp. PCC 6803	Ss_PCc_6803	Aquatic (Freshwater)	3.95	4	3610	61.77
Synechocystis sp. PCC 6803	Ss_PCC_6803	Aquatic (Freshwater)	3.95	7	3628	60.45
Synechocystis sp. PCC 6803, GT-I	Ss_PCC_6803_GT_I	Aquatic (Freshwater)	3.57	0	3217	65.12
Synechocystis sp. PCC 6803, PCC-N	Ss_PCC_6803_PCC_N	Aquatic (Freshwater)	3.57	0	3217	65.15
Synechocystis sp. PCC 6803, PCC-P	Ss_PCC_6803_PCC_P	Aquatic (Freshwater)	3.57	0	3218	65.13
Synechocystis sp. PCC 6803 (updateJune2012)	Ss_PCC_6803_U	Aquatic (Freshwater)	3.95	4	3771	59.29
Synechococcus sp. PCC 7502	Ss_PCC_7502	Aquatic (Freshwater)	3.58	2	3669	55.82
Acaryochloris marina MBIC11017	Am_MBIC11017	Aquatic (Marine)	8.36	9	8488	39.87
Candidatus Atelocyanobacterium thalassa ALOHA	CAt_ALOHA	Aquatic (Marine)	1.44	0	1241	67.77
Cyanothece sp. BH68, ATCC 51142	Cs_BH68_ATCC_51142	Aquatic (Marine)	5.46	4	5354	48.6
Nodularia spumigena CCY9414	Ns_CCY9414	Aquatic (Marine)	5.47	0	5363	46.93
Prochlorococcus marinus AS9601	Pm_AS9601	Aquatic (Marine)	1.67	0	1988	52.16
Prochlorococcus marinus marinus CCMP 1375	Pm_CCMP_1375	Aquatic (Marine)	1.75	0	1933	54.22
Prochlorococcus marinus pastoris CCMP 1986	Pm_CCMP_1986	Aquatic (Marine)	1.66	0	1766	59.29
Prochlorococcus marinus MIT 9211	Pm_MIT_9211	Aquatic (Marine)	1.69	0	1901	54.71
Prochlorococcus marinus MIT 9215	Pm_MIT_9215	Aquatic (Marine)	1.74	0	2059	51.09
Prochlorococcus marinus MIT 9301	Pm_MIT_9301	Aquatic (Marine)	1.64	0	1967	52.72
Prochlorococcus marinus MIT 9303	Pm_MIT_9303	Aquatic (Marine)	2.68	0	3133	41.94
Prochlorococcus marinus MIT 9312	Pm_MIT_9312	Aquatic (Marine)	1.71	0	1860	56.51
Prochlorococcus marinus MIT 9313	Pm_MIT_9313	Aquatic (Marine)	2.41	0	2334	54.2
Prochlorococcus marinus MIT 9515	Pm_MIT_9515	Aquatic (Marine)	1.7	0	1968	52.59
Prochlorococcus marinus NATL1A	Pm_NATL1A	Aquatic (Marine)	1.86	0	2251	47.09
Prochlorococcus marinus NATL2A	Pm_NATL2A	Aquatic (Marine)	1.84	0	1985	52.95
Rivularia sp. PCC 7116	Rs_PCC_7116	Aquatic (Marine)	8.73	2	6946	50.1
Synechococcus sp. CC9311	Ss_CC9311	Aquatic (Marine)	2.61	0	2951	47.88

Synechococcus sp. CC9605	Ss_CC9605	Aquatic (Marine)	2.51	0	2761	48.79
Synechococcus sp. CC9902	Ss_CC9902	Aquatic (Marine)	2.23	0	2410	53.61
Synechococcus sp. KORDI-100	Ss_KORDI_100	Aquatic (Marine)	2.79	0	3058	47.09
Synechococcus sp. KORDI-49	Ss_KORDI_49	Aquatic (Marine)	2.59	0	2717	51.27
Synechococcus sp. KORDI-52	Ss_KORDI_52	Aquatic (Marine)	2.57	0	2823	49.42
Synechococcus sp. PCC 7002	Ss_PCC_7002	Aquatic (Marine)	3.41	6	3237	58.67
Synechococcus sp. RCC 307	Ss_RCC_307	Aquatic (Marine)	2.22	0	2588	52.7
Synechococcus sp. WH 7803	Ss_WH_7803	Aquatic (Marine)	2.37	0	2591	53.69
Synechococcus sp. WH 8016	Ss_WH_8016	Aquatic (Marine)	2.71	0	3046	48.52
Synechococcus sp. WH 8109	Ss_WH_8109	Aquatic (Marine)	2.11	0	2713	45.3
Synechococcus sp. WH8102	Ss_WH8102	Aquatic (Marine)	2.43	0	2588	52.59
Trichodesmium erythraeum IMS101	Te_IMS101	Aquatic (Marine)	7.75	0	5156	41.64
